# The Contextual Adaptation and Digitization of an Online Parenting Program for Displaced Families: A Pilot Study With Latiné Immigrant Parents

**DOI:** 10.1111/famp.70091

**Published:** 2025-11-21

**Authors:** Jasmine M. Banegas, Tori S. Simenec, Lynn Muldrew, Melissa Uribe, Abigail H. Gewirtz

**Affiliations:** ^1^ Institute of Child Development University of Minnesota Minneapolis USA; ^2^ Reach Institute Arizona State University Arizona USA; ^3^ Paul Baerwald School of Social Work Hebrew University of Jerusalem Jerusalem Israel

**Keywords:** contextual adaptation, digital intervention, Latine families, parenting, prevention

## Abstract

Immigrant and refugee families experience heightened stress and trauma before, during, and after their migration journeys. In the last decade, research on parenting interventions for immigrant and refugee families has increased, inspiring researchers to explore effective, efficient, and sustainable methods that enhance service utilization and resilience. While trauma‐informed preventive interventions are available in the United States and other high‐income countries, accessible contextually and culturally appropriate interventions remain a challenge for forcibly displaced families. Considering the twin phenomena of 21st century globalization and digital technology, cultural adaptation and digitization of preventive interventions appear to be crucial to increasing access for families but overwhelming for interventionists to implement. Therefore, we present a step‐by‐step overview for researchers and family therapists to demystify the contextual adaptation and digitization process for cross‐cultural evidence‐based preventive interventions. This unique adaptation approach is intended to promote accessible programs developed for immigrant and refugee families from various cultures and with diverse migration experiences. The paper describes how a university‐based program development team applied cultural adaptation frameworks, community‐based participatory methods, and digital technology to contextually adapt and digitize Parenting in the Moment, the first digital trauma‐informed parenting program for displaced families in the United States. Researchers and clinicians may use and test this unique adaptation approach with other family‐based interventions.

## 1 Introduction

Globally, forced displacement has reached unprecedented rates, affecting approximately 40% of individuals under 18 (United Nations High Commissioner for Refugees [UNHCR] 2021). Over the last 10 years, research on parenting interventions aiming to reduce migration and resettlement stress has rapidly increased (Gillespie et al. 2022). However, the majority are delivered in person with limited reach; contextually and culturally appropriate support is inaccessible to most immigrant and refugee families (Gillespie et al. 2022; Simenec and Reid 2022). Thus, our team contextually tailored Parenting in the Moment (PIM), an evidence‐based trauma‐informed digital parenting program designed for diverse immigrant and refugee children and families. Although PIM is designed to support all families experiencing forced displacement to the United States, this paper describes the pilot program, outlines our cross‐cultural contextual adaptation process more broadly, and describes how we assessed whether Latines would consider the program relevant and acceptable.

### 1.1 Latino/a/e Children and Parents in the United States

Latines are the largest immigrant group in the United States. Recent increases in migration from Latin America include people from Guatemala, Honduras, El Salvador, and Venezuela seeking asylum due to economic instability, political persecution, organized crime, poverty, and large‐scale disruption from natural disasters (Batalova and Hoffman 2023; Duvillier et al. 2023). In 2018, nearly 19 million Latine children were living in the United States, comprising approximately one‐quarter of all U.S.A. children under 18 (National Research Center on Hispanic Children & Families NRCHCF 2020). Notably, 93% of Latine children are U.S.A‐born, reflecting their deep roots within U.S.A. mainstream society (NRCHCF 2020). However, these children often live in complex and diverse family contexts that shape their developmental experiences. Many grow up in mixed‐status families, with over half (53%) living with at least one foreign‐born parent and one‐fourth with a parent who is undocumented (NRCHCF 2020).

Previous research has demonstrated that protective factors, such as *familismo* (the importance and priority of family) and *respeto* (obedience and respect) are associated with positive parent and child outcomes. For example, *familismo* is related to decreased rates of child abuse (Coohey 2001), warm parenting (Gonzales et al. 2011), and less parent–child conflict (Kuhlberg et al. 2010), whereas *respeto* is linked to more cooperative behavior Kuhns and Cabrera (2020) and less risk‐taking (Velazquez et al. 2024). As Latine families become a larger segment of the United States population, it is crucial to understand the diversity, experiences, and needs of Latine families to inform prevention programs to support their mental health and wellbeing (Meléndez Guevara et al. 2021).

### 1.2 Are Contextual and Cultural Adaptations Worth It?

Prevention researchers have historically debated the tensions between implementing evidence‐based programs as intended versus tailoring them to meet the demands of diverse populations (Cabassa and Baumann 2013). One main challenge in adaptation research is the limited consensus on operationalizing adaptation (Wiltsey Stirman et al., 2013). *What* to adapt and *how* much to adapt frequently comes into question as developers and implementers work toward increasing a program's contextual and cultural relevance, which may impact program implementation, dissemination, and sustainability (Barrera et al. 2017).

Although contextual and cultural adaptations are interrelated and necessary to improve program fit, the two concepts emphasize distinct characteristics. Contextual adaptation involves tailoring an intervention according to the characteristics of a new setting that may change over time, including but not limited to geographic, service, cultural, economic, legal, and political contexts (Davey et al., 2018). Cultural adaptation, on the other hand, involves systematic tailoring to a target population according to language, culture, and context (Bernal et al. 2009). Thus, successful adaptation occurs when the tailored program increases engagement and perceived cultural acceptability while upholding the core components essential for effectiveness (Balci et al. 2022). Ultimately, effective adaptation balances flexibility with fidelity, assuring that interventions remain both contextually and culturally appropriate without compromising their effectiveness (Castro et al. 2004) to enhance acceptability and sustain positive outcomes among diverse populations with shared experiences.

For researchers and family therapists aiming to tailor programs for diverse populations in cross‐cultural settings, utilizing robust frameworks is essential for precise and accurate adaptation. Two theoretical frameworks focused on culture and context have historically informed intervention adaptation studies among prevention researchers. Resnicow et al.'s (2000) cultural sensitivity framework differentiates *surface‐* and *deep‐level adaptations*. Surface‐level adaptations are changes in program materials and activities to match characteristics of the intended audience (e.g., language), to enhance recipients' engagement. Deep‐level adaptations address complex cultural, social, or historical factors that impact recipients' experiences (Resnicow et al. 2000). A meta‐analysis focused on cognitive behavioral interventions for depression among Latines highlighted the significance of “surface‐” and “deep‐” level cultural adaptations across 8 randomized control trials and one quasi‐experiment, with evidence suggesting that deep‐level adaptations may increase intervention success rates between 15% and 30% compared to treatment‐as‐usual (Escobar and Gorey 2018). In line with Resnicow's conceptual framework, the Ecological Validity Model (EVM; Bernal et al. 1995) recommends using eight culturally sensitive dimensions (i.e., language, persons, metaphors, content, concepts, goals, methods, context) to ensure cultural and contextual responsiveness. These dimensions allow researchers to assess, identify, and organize cultural considerations that will inform a program's content and the methods used in the adaptation process (Simenec, Banegas, et al. 2023). Relatedly, a meta‐analysis by Soto et al. (2018) showed that two types of cultural adaptations based on Bernal's eight dimensions were significantly associated with improved treatment outcomes: (1) aligning treatment goals with recipients' cultural values and (2) offering services in recipients' preferred language.

The Cultural Adaptation Process Model (CAPM) is a three‐phase conceptual framework that has been applied to tailor family‐based programs for diverse ethnic populations (Baumann et al. 2014; Domenech‐Rodríguez and Wieling 2004). CAPM extends the foundational work of Resnicow and Bernal by placing the community at the heart of implementation and dissemination initiatives. Phase 1 focuses on collaboratively setting the stage, gathering information, and identifying three key personnel to support program adaptation (e.g., the change agent (university‐degreed professional with technical program expertise), opinion leader (community expert), and members of the community) (Domenech‐Rodríguez and Wieling 2004). Phase 2 focuses on conducting preliminary program adaptation based on initial feedback and consultation with community leaders and members. The final phase considers adaptation iterations to augment accurate program refinements (Domenech‐Rodríguez and Wieling 2004). Overall, CAPM is designed to provide a clear *how‐to* practical guide that outlines the general process of intervention adaptation through community‐engaged collaborations.

### 1.3 Systematic Documentation of Intervention Adaptations

In light of the limited general evidence on the systematic reporting of contextual and cultural adaptation methods (Spanhel et al. 2020), experts in the field are calling for published reports that detail decision‐making and implementation processes to boost transparency and replicability (Heim et al. 2021). This call has led implementation researchers to develop documentation tools, such as the Framework for Reporting Adaptations and Modifications to Evidence‐Based Interventions (FRAME) taxonomy to systematically categorize the nature, timing, and reasons for modifications, and maintain fidelity in real‐world contexts (Wiltsey Stirman et al. 2019). Previous research shows that FRAME has been used to tailor interventions in new settings (Madrigal et al., 2023) and for new populations (e.g., Jang et al. 2022; Parker et al. 2022). The FRAME model is noted to be promising for implementation teams because of its potential to enhance the feasibility of adaptations and facilitate their program evaluation to empirically measure treatment outcomes (Parker et al. 2022).

### 1.4 Digital Interventions for Immigrant and Refugee Families

Utilizing digital platforms to deliver parenting programs tailored for immigrant families can increase access to care, address barriers like money and time, and offer culturally and contextually informed resources (Soltero‐González and Gillanders 2021). Digital programs may increase the scalability and accessibility of key resources for families. Even though rigorous outcome evaluations of digital parenting interventions for immigrant and refugee populations are just beginning, a review including 16 digital health interventions for immigrant populations indicated general user satisfaction (Liem et al. 2021). However, the review also noted the importance of digital literacy in supporting the scalability of digital services in immigrant and refugee communities. Although globalization has led to a worldwide increase in technology adoption, digital inequities, characterized by disparate utilization of existing technological resources and infrastructure remain a significant barrier (Kuhn et al. 2023). From an implementation standpoint, digitization offers increased ease of adaptability compared to traditional in‐person programs allowing for efficient, cost‐effective, tailoring to match the target population's context and worldview (Simenec, Gillespie, et al. 2023).

### 1.5 After Deployment, Adaptive Parenting Tools (ADAPT Program)/ADAPTonline

GenerationPMTO/Parent Management Training Oregon Model (Patterson 1982, 2005) is based on the social interaction learning theory and has served as the foundation for the adaptation of various parenting programs (Ballard et al. 2017; Baumann et al. 2014); Forgatch and DeGarmo (2011). Its core skills—encouragement, effective discipline, monitoring, problem‐solving, and positive involvement—are designed to strengthen parent–child relationships and increase children's prosocial development (Patterson 1982, 2005). The After Deployment Adaptive Parenting Tools (ADAPT) program is the first adaptation of GenerationPMTO that integrates emotion socialization as a core skill and has been rigorously tested in multiple formats to meet the needs of military families with school‐aged children facing deployment stress (Gewirtz et al. 2011; Gewirtz et al. 2024).

ADAPTonline, a self‐paced online program, includes 14 skill‐building modules, each with a brief (5–10 min) skill video, practice video, mindfulness practice, reflective questions, and handouts (Gewirtz et al. 2024). Video content shows ineffective and effective parenting strategies, ways to practice, and testimonials from former participants (Gewirtz et al. 2024). ADAPTonline's six core skills are teaching through encouragement, effective discipline, monitoring, problem‐solving, positive involvement, and emotion socialization. Although the original GenerationPMTO model was not developed as a trauma‐informed intervention, ADAPT considers the widespread impact of deployment trauma on military families and seeks to support their path toward positive adaptation through effective parenting and emotion regulation strategies. Contextual modifications of ADAPT include military‐specific metaphors, examples, and parenting vignettes (Gewirtz et al. 2011).

### 1.6 Parenting in the Moment: A Pilot Study

Building on ADAPTonline, Parenting in the Moment (PIM) is the first trauma‐informed digital parenting program for immigrant and refugee families resettled in the United States. Considering that immigrant and refugee families experience trauma across the migration spectrum (Perreira and Ornelas 2013), a trauma‐informed program is necessary to increase parents' understanding of how stress may impact parenting efficacy and children's development. Therefore, PIM focuses on the context of migration and resettlement stress experienced by immigrant families more broadly and also considers specific cross‐cultural adaptations that may be acceptable with specific ethnic groups. Particularly, the Spanish version of the program was further culturally refined using surface‐level adaptation to increase relevance among Latine families. Although PIM was not developed only for Latine families, the goal of this paper is to focus on the acceptability of the program among Latine families. Therefore, this paper will first describe PIM's step‐by‐step community‐engaged contextual adaptation process guided by the Cultural Adaptation Process Model (CAPM; Domenech‐Rodríguez and Wieling 2004). We will also draw on the Framework for Reporting Adaptations and Modifications to Evidence‐Based Interventions (FRAME; Wiltsey Stirman et al. 2019) to document the timing, nature, and reason for PIM's modifications and the Ecological Validity Model (EVM; Bernal et al. 1995) to interpret the contextual and cultural relevance of these adaptations.

## 2 Method

### 2.1 Contextual Adaptation and Digitization Procedures

**FIGURE 1:**
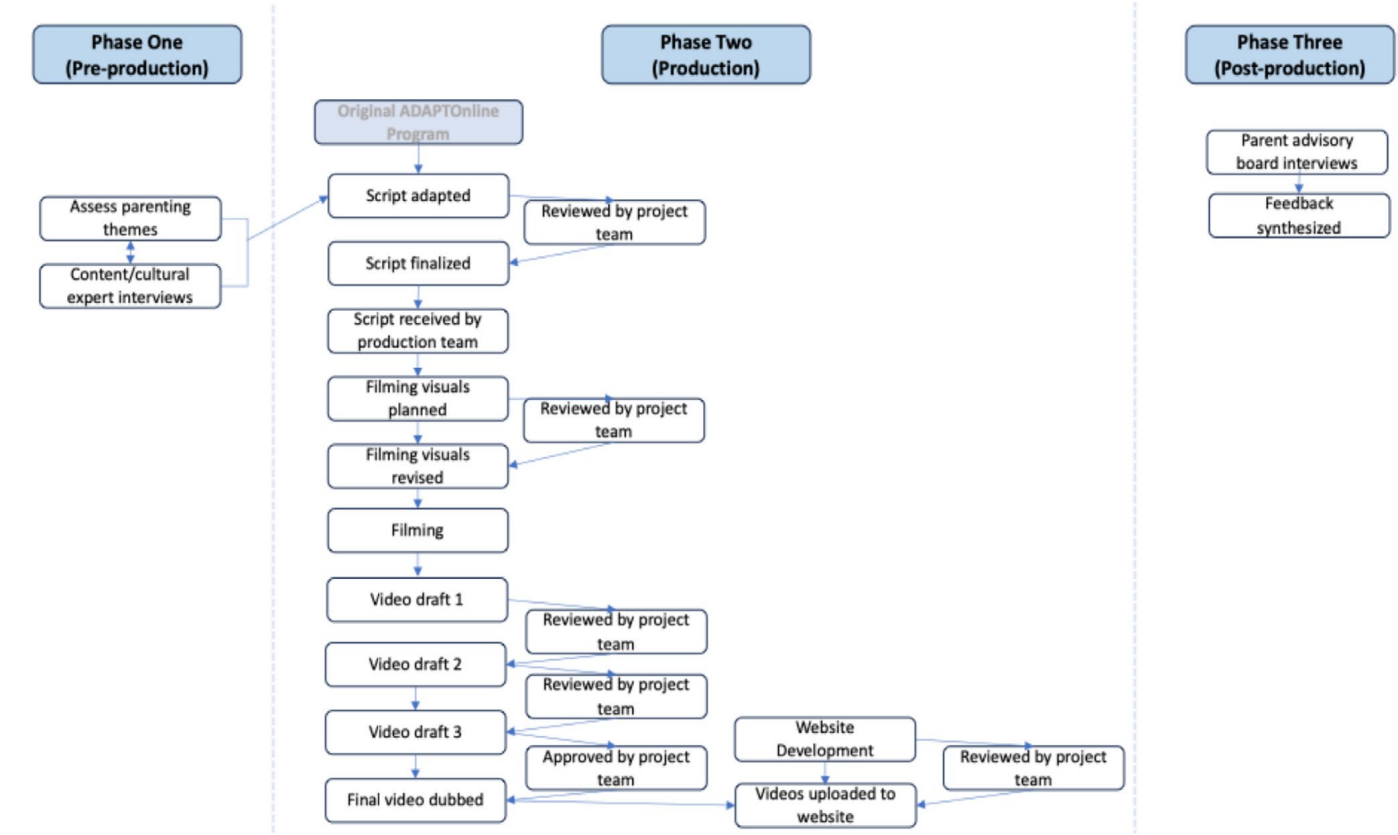
PIM's contextual adaptation and digitization process flow.

The contextual tailoring of PIM for immigrant and refugee families began in August 2019 (see Figure 1 for adaptation process flow). The current pilot study was conducted in three community‐engaged phases guided by the CAPM (Domenech‐Rodríguez and Wieling 2004): (a) pre‐production/information gathering with diverse immigrant populations to generate cross‐cultural parenting themes, (b) production/PIM's initial contextual modifications, and (c) post‐production/iterative PIM content modifications informed by Latine community members. The interdisciplinary program development team included experts in prevention/intervention, parenting, trauma, marketing, and film. The program development team included the principal investigator (PI; developer of ADAPT and a certified GenPMTO mentor), clinical director (certified GenPMTO trainer and coach), and Spanish‐speaking cultural adaptation specialist (CAS; certified GenPMTO facilitator and trainer). To ensure that PIM remained true to ADAPT's core components, the PI and clinical director provided immediate consultation and reviewed PIM scripts adapted by the CAS from the ADAPT scripts. Adaptations extended beyond the online program to include marketing resources; see Simenec, Banegas, et al. (2023) for more information. At the time of initial implementation, PIM's pilot project was not intended for research dissemination. In consultation with the University of Minnesota's Institutional Review Board, the project was determined to be a program development/quality improvement activity and not human subjects research, and formal IRB review was not required.

### 2.2 Phase One (Pre‐Production): Information Gathering and Cultural Expert Interviews

While there is no singular process for cultural adaptation, many cultural adaptation studies use community‐based participatory methods to ensure that community voices are represented throughout the tailoring process (Archer‐Kuhn et al. 2022). Therefore, the pre‐production phase involved three complementary steps to gather insights from community members to lay the groundwork for program development.

Step 1: Identify a cultural insider to serve as the contextual adaptation specialist and review relevant literature. First, the team recruited a Latina bilingual Spanish‐speaking certified ADAPT facilitator to serve as the CAS. The program development team reviewed literature on cultural adaptation of parenting programs and immigrant/refugee mental health in the United States to identify key immigrant/refugee populations for PIM and select appropriate cultural adaptation frameworks to inform the contextual and cultural tailoring methods.

Step 2: Build relationships with cultural experts and conduct needs assessment. Given the broad emphasis of PIM, the CAS met with 5 cultural experts to speak to specific community needs. Cultural experts recruited were licensed mental health providers, researchers, and a state Department of Human Services clinical consultant. They were from diverse regions of the world, such as Iraq, Ethiopia, Somalia, Mexico, and Colombia. Cultural experts' involvement was extensive including, for example, sharing their personal stories to inform instructional video development, capture the context of migration and resettlement, and identify common cross‐cultural themes that would be acceptable to multiple ethnic groups.

Step 3: Assess parenting themes among immigrant and refugee families to inform adaptation. The program development team launched a 12‐item anonymous online survey with open‐ended questions through Amazon's Mechanical Turk/MTurk digital crowdsourcing platform, to identify broader parenting themes among English‐speaking parents who experienced forced migration to the United States and had 4–12‐year‐old children. MTurk was chosen due to the opportunities to efficiently collect information that may be generalized across diverse people and contextualized to fit specific groups of people, settings, and times (Zhu et al., 2015). The program development team asked parents to reflect broadly on their parenting experiences before and after coming to the United States, offering participants ~US$2.50 as a small token for their time. Sample questions included, “what values or ideas are important to you and your family?”, “what are your hopes for your children?”, and “what are the main parenting challenges you have experienced since coming to the United States?” After 21 days, we received a total of 198 responses, but only 41 participants were included due to missing or low‐quality data. Survey respondents were from Latin America (Colombia, Mexico, Belize, Venezuela, Cuba, El Salvador), the Middle East (Yemen, Iraq, Syria), Africa (Ethiopia, Kenya, Liberia), Central Asia (Afghanistan, Pakistan), and Southeast Asia (Burma, Bangladesh, Vietnam, China).

The crowdsourcing approach allowed the program development team to informally code responses and generate broad cross‐cultural themes to highlight various parenting skills, challenges, values, and goals while taking into account the context of forced migration. With the support of one research assistant, the CAS utilized an analytical memo writing approach to track the frequency of common words mentioned by participants and assist with the reflection of open‐ended survey results (Birks et al. 2008). Upon completion of our review, the top three values or ideas identified as being important for these parents were “family,” “morals,” (respect, honesty, and responsibility) and “education” (hopes for children to receive a good education and be successful in this new country).

### 2.3 Phase Two (Production): Initial Contextual Adaptation and Digitization of PIM

Phase two encompassed two key activities: a priori tailoring of the program (Domenech Rodríguez & Bernal, 2012) and its digitization. A priori adaptations were made using the information collected in phase one and integrating the Ecological Validity Model framework (EVM; Bernal et al. 1995). The program development team considered digitization practices to maximize program attractiveness before the implementation rollout.

**FIGURE 2:**
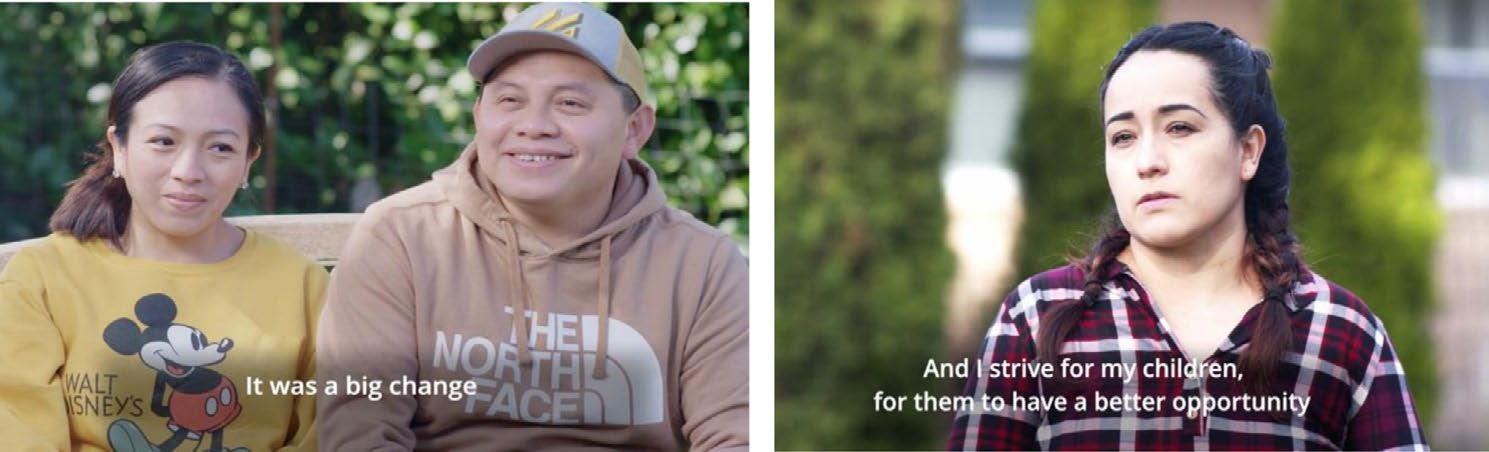
Latine parents storytelling (After Adaptation); Illustrations of PIM Module One. Indigenous couple from Mexico, Yucatan, Mayan Community (left); Mother from Mexico (right).

Step 4: Preparing the program video scripts. Following the original ADAPTonline video script structure, each PIM video involved scenarios of ineffective/effective parenting strategies, skill explanations, and reflection activities. Given that ADAPTonline was contextually tailored to meet the needs of military families in English, our team identified surface‐level changes (See Figure 2) to maximize acceptability across immigrant and refugee communities, including those from Latin America. Using a video script template, the CAS created a “blueprint” (Simenec, Gillespie, et al. 2023) by color coding standard video visuals and embedding contextual and culture‐specific considerations needed to reflect the diversity of immigrant and refugee populations and their experiences (Table 1). Contextual modifications emphasize broad stressors immigrant and refugee families face during resettlement, such as access to basic needs, fear of anti‐immigrant sentiment, parent–child acculturation conflicts, and limited community, family, and financial support (Parra Cardona et al. 2012). Culturally specific surface‐level changes included language, names, clothing, and foods.

**TABLE 1:** PIM video script template: Building blocks of resilience (Module Two).

Scene	Narration & Dialogue	Visual
1	*Parenting in the moment*	Parenting in the Moment logo [GRAPHIC]
2	NARRATION: Call to action recommending participants to watch videos in order to give the nature of the skill‐building techniques.	Building Blocks of Resilience [GRAPHIC]
3	CONTEXTUAL NARRATION: Description of PIM	Montage of positive family interactions with diverse parents [ACTION]
4	NARRATION: Probing questions for viewers surrounding parenting values.	Incorporating an immigrant family sharing a moment together [ACTION]
5	DIALOGUE: Immigrant/refugee parents describing their hopes for their children and how they strengthen their relationships with their children	Series of immigrant/refugee parents firsthand accounts [ACTION]
6	NARRATION: Offering definition and explanation of “Parenting Values” using simpler terms for English language learners.	Incorporating family values that may resonate with immigrant/refugee populations [GRAPHIC]
7	DIALOGUE: Diverse immigrant/refugee parents talk about their various values and the ways they embed them in their children while living in a new country.	Interviews with immigrant/refugee parents [ACTIONS]
8	NARRATION: Encourage viewers to write down some their values. Closing statement and a sneak peek of the next skill.	Call to action for home practice. [GRAPHIC]

*Note:* Blue, Reference to module's key skill; Red, Contextual consideration.

Once the video scripts were created, a second round of interviews with Latine cultural experts was coordinated to gather additional feedback on the program's content and how the program effectively demonstrated the target audience's diversity. The program development team synthesized cultural expert insights and incorporated experts' feedback into the videos.

Step 5: Digitizing PIM's video content. The team worked with a production company to develop the video content including finding locations, and actors from immigrant and refugee communities represented in PIM. The skill videos were filmed in diverse settings, including parks and homes (e.g., bedrooms, living rooms, kitchens, yards) to depict a context that would resonate with program participants. The videos included commentaries delivered by a narrator and immigrant parent actors, including Latine parents, to demonstrate how to use the parenting skills taught in the program. Stories were first‐hand accounts from Latine and other diverse immigrant parents, as well as cultural experts to normalize the challenges immigrant and refugee families experience (Kia‐Keating and Juang 2022). The CAS and videographer prepared interview questions for cultural expert endorsements that were aligned with PIM skills. During editing, the program development team assessed each iteration of video revisions to ensure the correct use of subtitles, contextually appropriate animations and graphics, good sound quality, and narration.

Upon completion of the English version, the four PIM modules and accompanying graphics were professionally dubbed into Spanish and back‐translated by the CAS to ensure that the intended psychoeducational objectives were accurately conveyed. Similar to the English language process, word choices were identified by the cultural experts and discussed during program development meetings (see Table 2). An example of language consideration was the selection of a Spanish dialect that could be understood by most Spanish speakers in the United States and translated into text. Advisory group discussions with Latine parents helped to refine PIM Spanish metaphors.

**TABLE 2:** Community feedback synthesis according to EVM domains.

*EVM Domains*	Video 1: Welcome to PIM	Video 2: Values and Goals	Video 3: Values and Goals	Video 4: Teaching Positive Behavior
Language	Include dubbing/subtitles for accessibility. Avoid “refugee” vs. “immigrant” to include undocumented families. Used clear and engaging language (e.g., “journey”, “best teacher”) Accents of parents and expert make it relatable. “Our English is not perfect.” Careful translation to ensure intended meaning.	Language is simple and accessible in English and Spanish. Explanation of values and goals are clear and well‐received.	Language is simple and accessible in English and Spanish. Suggest replacing “cooperation” with culturally inclusive terms like “connection” or “understanding.”	Words like “qualify” and “praise” are abstract; may not be easily understood. Most language is clear, but adjustments could enhance comprehension further.
Persons	Diverse representation well‐received; promoted relatability.	Diverse representation well‐received; promoted relatability.	Diverse representation well‐ received; promoted relatability. Parenting focus ensures relevance across SES and migration stories	Diverse representation well‐received Families sharing experiences with cooperation and encouragement strengthens relatability.
Metaphors	Healthy eating metaphor effectively illustrates parenting cross‐culturally. Highlighting intuition affirms practices common in displaced communities.	Integrating values from both countries of origin and the U.S.A. found relatable. Incorporating universally resonant values (e.g., family, morals, and respect) suggested.	Effective/Ineffective directions scenarios are broad and general. Culturally specific metaphors are not needed; cross‐cultural relevance is strong and impactful.	Mother encouraging child resonated with families. Strong visuals and metaphors (e.g., flower analogy, shining the light, 5:1 rule); highly impactful. Emphasizing differences in discipline between home and host countries to enhance relatability.
Content	Content becomes universally relatable when it is parent‐focused.	Integrating values from origin and U.S.A. culture was relatable. Clear illustration of effective/ineffective goal setting helped parents. Idea of biculturalism could be expanded. Broaden values and goals for cross‐ cultural audience. Definition of parenting values for clarity.	Addresses giving effective directions, linking compliance and emotion regulation. Focus on universal parenting skills enhances cross‐cultural resonance. Narrowing objectives boosts impact.	Effectively explains encouragement/cooperation with phrases like “shine the light”/ “focus on positives.” Depicts parenting skills with cross‐ cultural relevance (e.g., diverse scenarios and cultures) Showing respect and using positive parenting align with community values.
Concepts	Introduction highlights PIM's relevance for diverse migrant parents. Focusing on parenting ensures inclusivity across varied migrant experiences.	Clearly explains values and goals. Read aloud written text for accessibility. Use images to highlight values (i.e., education = youth graduating).	Provides definitions, role‐plays, and examples of parenting skills. Number skill steps for accessibility. Demonstrates strong content delivery and teaching methods.	Models key concepts with effective/ineffective examples. Visual aids effectively demonstrate concepts. Cross‐cultural relevance is strong as the video centers on parenting.

*Note:* EVM Ecological Validity Model (Bernal et al. 1995).

Step 6: Developing PIM's website and uploading video content. Simultaneous with Steps 4 and 5, the program development team worked with a software company to create a website for parents to access PIM's digital content. The team used the Vimeo platform to host and track the number of views of each video (Simenec, Banegas, et al. 2023). The program development team collaborated with web designers and a marketing team to design culturally responsive messaging for PIM's website. Sample website verbiage emphasized helping immigrant parents to “make every moment count when it comes to supporting your family to thrive in a new country” (parentinginthemoment.org). After development, members of the team reviewed the website's Spanish translation to ensure accuracy and tested the features and functionality using both computers and smartphones. The internal usability testing process allowed for troubleshooting of technical problems well in advance of facilitating focus groups to gauge acceptability and perceived usability with parents.

### 2.4 Phase Three (Post‐Production): Adaptation Iterations With Intended Recipients

PIM's post‐production phase focused on making further contextual and cultural changes as necessary to ensure the continued relevance, appropriateness, and generalizability of the program's content and tailoring process. To make changes, the program development team sustained community‐based participatory efforts to receive feedback from intended recipients.

Step 7: Facilitating parent advisory group discussions. We recruited three bilingual Latine (Mexican)^1^ immigrant parents of 4–12‐year‐old children for the parent advisory group. Parents were informed that PIM aims to highlight the diversity of migration experiences and thus, they were encouraged to consider contextual and cultural nuances that would be relevant and acceptable to both Latine and non‐Latine immigrant communities. The parent advisory group watched the four PIM videos at home in (English)^2^ filled out an EVM‐informed worksheet (See Table S1) and met with the CAS and a graduate student to share feedback, using a bottom‐up approach to brainstorm ideas for the graphics, metaphors, and scenarios. Parents were compensated US$100.

Step 8: Synthesizing Community Feedback and Generating Themes. The program development team organized the feedback from the parent advisory group discussions and cultural experts using the eight dimensions of the EVM (See Table 2). This was used to guide the team's understanding of how to reflect the migration context more broadly. Suggestions for modifying text, storyline, illustrations, and reasons for tailoring were recorded. The program development team met weekly to discuss and align modifications with PIM's theoretical model, social interaction learning (Patterson 1982, 2005), which emphasizes the role of positive parenting to promote resilience and reduce coercion in the forced migration context.

## 3 Results

### 3.1 Documented Contextual and Cultural Adaptations

**FIGURE 3:**
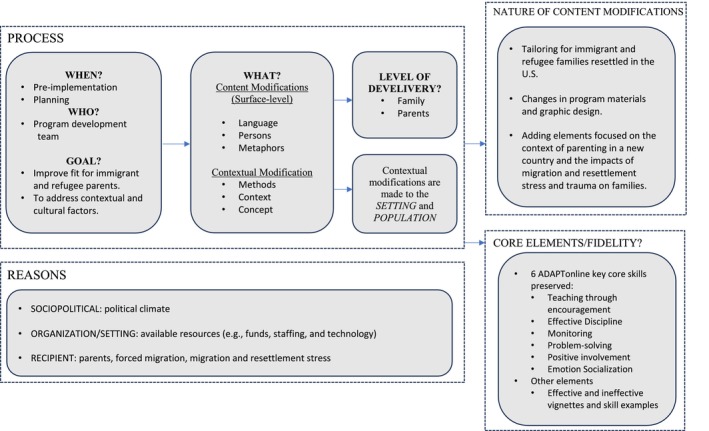
Timing, nature, and reasons for adaptation based on the FRAME documentation tool. FRAME: Framework for Reporting Adaptations and Modifications to Evidence‐Based Interventions (Wiltsey Stirman et al. 2019).

In this section, we describe the timing, nature, and reasons for PIM's modifications using the FRAME (Wiltsey Stirman et al. 2019). As shown in Figure 3, PIM's modifications occurred during the pre‐implementation/planning phase of this study. The nature of the adaptations implemented emphasized (a) content changes to intervention materials, examples, and framing; and (b) contextual changes to fit the local context and increase the program's relevance among immigrant families more generally (Wiltsey Stirman et al. 2019). The program development team preserved the original core elements of ADAPTonline, including 6 key skills (e.g., encouragement, effective discipline, monitoring, problem‐solving, positive involvement, and emotional socialization).

We also apply the EVM model dimensions below to interpret, illustrate examples of changes, and highlight the insights derived from cultural expert and parent advisory group discussions (Table 2). For this pilot, culturally specific surface‐level adaptations were made to enhance the receptiveness among Latine parents. Deep‐level cultural adaptations were not made to ensure applicability across diverse cultures.

1. ***Language***, as defined in the EVM, must be contextually appropriate, culturally informed, and comprehensible (Bernal et al. 1995; Domenech Rodríguez et al. 2011). To address this dimension, general language modifications included basic descriptions of the program's concepts with paced captions and simple terms for English language learners. Although most of the cultural experts and parent advisory group all shared that the English language was clear, simple, and easy to understand, some suggested avoiding the use of the terms “immigrant” versus “refugee” throughout the program to avoid unintentional exclusion of undocumented families. Other recommendations made included replacing “cooperation” with culturally inclusive terms like “connection” or “understanding” to reflect the common collective cultural values among immigrant communities. Culturally specific modifications involved common Latin American words and phrases for different program concepts, such as “valores y objetivos” (values and goals), “*elogiar*” (to praise), and “formas de estar presente” (ways to be in the moment/present). These were reported to be clear and comprehensible.
2. The ***people*** dimension refers to ensuring racial, ethnic, and cultural match between the intended recipient (i.e., those exposed to forced migration) and the program development team (Bernal et al. 1995). Accordingly, general modifications included actors and interviewees who were immigrant parents of a school‐aged child and from diverse regions (e.g., Latin America, the Middle East, and Africa). The parent advisory group appreciated the broad representation of people in the videos because it made them feel that the program normalized parenting challenges for all immigrant families. Culturally specific modifications included common Spanish‐language names (e.g., Mila and Esmeralda/“Esme”) for Latine characters. Parents shared that the names were appropriate and representative of popular names in Latin America.
3. ***Metaphors*** capture the integration of symbols and concepts that are shared by the intended population (Bernal et al. 1995). Consistent with this dimension, general modifications involved ensuring cornerstone program metaphors (e.g., “shine the light on what you want to grow” “enfocar la atención en lo que quieras que florezca*”*) was easily understood and relatable to the intended audience (Table 2). For example, Latine parents shared that this reminded them of the Spanish‐language “dicho” or idiom, “arbol que crece torcido jamás su tronco endereza*”* (a crooked tree will never straighten its trunk). The relevance of comparing a tree or flower to the growth of a person resonated with cultural experts and the parent advisory group. Further, the comparisons emphasizing intuition to affirm practices common in immigrant communities, integrating bicultural parenting values, and acknowledging the differences in discipline between two cultures were viewed as strong and highly impactful.
4. ***Content and Concepts*** refer to broad knowledge related to local parenting values, customs, and traditions (Bernal et al. 1995); and the idea that PIM learning domains should be consistent with the intended population's cultural experience to ensure that program terms are relevant and clear (Bernal et al. 1995). The PIM videos include parent narratives, parenting scenarios, characters, and illustrations, making them an essential part of this skills‐based and scenario‐focused digital preventive intervention.

In line with these two EVM dimensions, narratives included stories from immigrant parents with diverse cultural backgrounds, from Mexico, Iraq, and the Democratic Republic of Congo. Videos focused on parents' experiences, feelings, and concerns about adjusting to a new country, and their parenting goals (Figure 2). Members of the parent advisory group reported feeling connected to the stories given the relevance to their day‐to‐day experiences as parents in a new country. The narratives heightened parent identification with PIM's content and overall objectives. Cultural experts noted that first‐hand accounts are essential elements of PIM's program content to help build mutual trust and increase program acceptability and engagement. Particularly, PIM's content was viewed as universally relatable due to its parent‐focused nature (Table 2).

Likewise, general modifications included parenting scenarios showcasing different family compositions (single‐ and two‐parent households, a range of children). Parents and cultural experts resonated with scenarios that illuminated the challenges and benefits of raising bicultural children in the United States (e.g., “parents are different here”, “I don't care what country you are in”, older siblings helping with child‐rearing). They also appreciated the demonstration of ineffective and effective parenting strategies, which are key aspects of the PIM curriculum.

**FIGURE 4:**
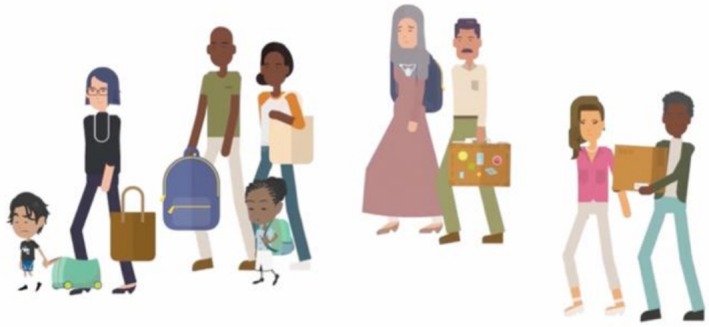
Animation representing multicultural immigrant and refugee families (Module One).

Culturally specific modifications highlighted values of obedience and respect. One parent reported that the Latine father depicted in the *ineffective parenting* scene for module 3 (teaching effective directions) did not appear “angry” enough. She asked that the father show “bigger” emotions—a louder, sterner voice, and more gestures. Our team re‐filmed the ineffective scenario, amplifying the father's frustration through tense body language, increased voice volume, and exaggerated gestures. When parent feedback did not align with PIM's core principles, the program development team co‐designed solutions with parents and experts that integrated PIM's evidence‐based strategies with contextually and culturally specific approaches to ensure program fidelity. For example, some parents shared concerns that rewarding their children for expected behaviors may undermine their values of respect and responsibility (see Table 2). Collaboratively, we identified culturally meaningful alternatives, such as words of affirmation and tokens, to preserve PIM's emphasis on teaching through encouragement and making it relevant for families. Moreover, the final graphics and animations were co‐created with the program development team, parent advisory group, and cultural experts. The graphics and animations varied depending on the cultural group in the video (Figure 4), and parent advisors shared that facial expressions, dress, and text elements of animated characters were realistic and relatable.

5 ***Goals*** involve ensuring that program aims and parent needs are congruent (Bernal et al. 1995). In response to the goals dimension, general modifications included depicting parents' hopes and dreams, such as “a good education” and successfully raising children in a bicultural context. Notably, in Latine families, “good education” (*”buena educación”*) represents having good morals like being willing to help others, kind, and family oriented (Cortes Barragan & Meltzoff, 2025). Thus, cultural experts reported that addressing bicultural integration (Bacallao and Smokowski 2009) was important for families served by PIM.
6 ***Contextual modifications*** emphasize ecological and sociocultural fit for the intended recipient (Bernal et al. 1995; Davey et al., 2018). An important aspect of PIM is acknowledging the immigration context more broadly because of the shared migration and resettlement experiences families face. Reflecting this dimension, general modifications included highlighting common parenting experiences, the diversity of migration experiences, the impact of migration and resettlement stressors, and the hopes and dreams of immigrant parents. Cultural experts agreed that modules also effectively represented the bicultural context in which children of immigrants are being raised. Cultural experts and parents agreed that the values (e.g., family, morals, and respect), visuals, language, and narration fit the local resettlement context, making it appropriate for Latine and other immigrants.

## 4 Discussion

Multiple evidence‐based parenting programs have now been adapted or digitized for online delivery (Florean et al. 2020). However, the vast majority are aimed at mainstream populations in the Global North (e.g., DuPaul et al. 2018; Sanders et al. 2012) with a notable exception (Parenting for Lifelong Health, e.g., Cluver et al. 2018). To our knowledge, PIM is the first digital parenting program for those forcibly displaced to the United States. Applying the Ecological Validity Model (Bernal et al. 1995) and a three‐phase cultural adaptation process (Domenech‐Rodríguez and Wieling 2004), this paper outlined the steps to adapting an evidence‐based online parent program for diverse immigrant communities. Though the program's focus is on families affected by forced migration from around the world, we focused on attaining detailed input from Latine parents to pilot the materials in English and Spanish. The goal of this process was to evaluate whether a broadly focused digital parenting program (with videos showcasing immigrants and refugees from different regions) would nonetheless be seen as relevant, acceptable, and comprehensible to Latine families.

The program's core components—the six key parenting skills tested in our ADAPTonline program—were retained in the development of PIM. The FRAME offered the opportunity for the program development team to systematically document the nature, timing, and reasons for PIM's content and contextual adaptations (Wiltsey Stirman et al. 2019). The adaptations made focused on the context of forced migration and the values, customs, traditions, and expressions of the families in immigrant communities. This highlights the crucial importance of a transdisciplinary team in the development of online programs for broad audiences with shared experiences including a skilled film production and web design team working closely with mental health professionals and cultural insiders to inform program development. Moreover, the strong community partnerships that enabled discussions with cultural experts, Latine parents, researchers, and providers were also essential during the adaptation and digitization of PIM.

### 4.1 Implications for Research and Practice

The current paper offers a blueprint (Simenec, Gillespie, et al. 2023) for the adaptation and digitization of evidence‐based parenting programs for culturally diverse immigrant families. Such contextual adaptations offer family therapy and other mental‐related fields accessible and acceptable preventive interventions to support families typically excluded from traditional service models.

#### 4.1.1 Research

As demonstrated in this study, leveraging cultural adaptation and implementation science frameworks may help researchers systematically document, contextualize, and interpret the acceptability of interventions for diverse populations. PIM's modifications primarily focused on contextual modifications to ensure the program's broad relevance among immigrant families resettled in the United States, while increasing acceptability among ethnic subgroups through surface‐level cultural modifications. Future clinical trials may also draw on implementation science to address emerging questions as they relate to identifying multisystem implementation barriers and facilitators (e.g., individual, family, organizations, and policies) and their impact on intervention goals.

The adaptation process findings from this pilot also highlight the importance of additional research to examine interactions between context, culture, and digital prevention programs for displaced populations. However, the digital divide means that even digital interventions are less likely to reach those typically excluded from research or with limited access to healthcare. To address digital inequities, researchers and program developers should work closely with program users to understand the role of culturally responsive marketing (Simenec, Banegas, et al. 2023), user‐friendly designs, supportive infrastructures (e.g., access to devices and connectivity), and educational support from implementers (Wilson et al. 2024). Co‐designing and co‐evaluating digital programs with community members may increase engagement among underserved communities.

#### 4.1.2 Clinical Practice

Digital programs like PIM offer family therapists and other clinical professionals ways to improve the delivery of services and help immigrant families overcome service barriers via mobile devices, web‐based platforms, or social media (Simenec, Banegas, et al. 2023), providing a gateway to in‐person intervention. Family therapists and other clinical professionals working with displaced families can use digital interventions to enhance in‐person and telehealth treatment experiences (Doss et al. 2017). Technologies such as PIM's skill video clips may be integrated into sessions to help therapists teach parents. Family therapists and other clinical professionals may also recommend digital parenting programs as asynchronous tools to remind parents of therapy skills and homework assignments outside the therapeutic space (Liverpool et al. 2020; Staiano et al. 2021). Integrating digital technology with in‐person services can improve quality access to care.

### 4.2 Limitations

The contextual adaptation process was lengthy and resource‐intensive, limiting its applicability to settings in which resources are relatively scarce. Second, much of the work described in this pilot was conducted during the COVID‐19 pandemic, which limited our recruitment of the parent advisory group, as well as our ability to meet in person, and it is possible that feedback would have been different with in‐person interactions. Moreover, the MTurk survey from phase one and the parent advisory group discussions from phase three were administered in English, which may have limited the representativeness of the sample given that many immigrant parents in the United States may not speak English. Last, while research in evidence‐based practices for families affected by forced migration advances, strategies to successfully facilitate the wide implementation of these interventions are needed. Although this pilot study reflects early iterations of PIM's adaptation process, and thus may not capture the full spectrum of possible modifications, the program development team values an iterative approach to further refine cultural fit and promote long‐term sustainability.

## 5 Conclusion

This study is the first to describe an approach to contextual and cultural adaptation of a digital parenting program for forcibly displaced families, adding to the literature highlighting the value of cultural and contextual adaptation for underserved communities. Steps remain to ensure that the PIM program is feasible, accessible, acceptable, and effective for a broad range of families exposed to forced migration. The full version of the program will be available in multiple languages (English, Spanish, French, and Arabic). We replicated the feedback process and conducted focus groups with parents from French‐ and Arabic‐speaking countries in Africa and the Middle East as well as those from Spanish‐speaking Latin American countries (Muldrew et al. 2023). A federally funded randomized controlled trial of PIM is underway.

## Conflicts of Interest

The authors declare no conflicts of interest.

## Supporting information


**Table S1:** EVM‐Informed Worksheet: Introduction (Module One).

## Data Availability

The data that support the findings of this study are available from the corresponding author upon reasonable request.

## Endnotes

^1^
In part because the group was formed during the COVID‐19 pandemic, our capacity to recruit as broadly as we would have preferred (i.e., immigrant parents originating in diverse Latin American countries) was limited.

^2^
PIM's Spanish language dubbing process had not yet been finalized at the time of the parent advisory group review; the dubbing process occurred after final edits were incorporated based on parent feedback.
