# Supplementary material for: The Contextual Adaptation and Digitization of an Online Parenting Program for Displaced Families: A Pilot Study With Latiné Immigrant Parents
Source: Fam Process. 2025 Nov 21;64(4):e70091. doi: 10.1111/famp.70091 (PMC12639195; doi:10.1111/famp.70091)
Supplement: Supplementary file 1 — Table S1: EVM‐Informed Worksheet: Introduction (Module One). [file FAMP-64-0-s001.docx]

**Supplemental Material**

**Table S1**

*EVM-Informed Worksheet: Introduction (Module One)*

| Topic | Questions to Consider |
| --- | --- |
| Language | - Is the language easy to understand? |
| People | - Do the actors in the videos resonate with you and those from your community? Why or why not? - Is there anything about the appearance of the actors that might cause parents in your community to not relate to the videos? |
| Metaphors | - Are the scenes relevant to you and other parents from your community? Why or why not? |
| Content | - Is the narration or voiceover clear, respectful, and easy to understand? Why or why not? - Are there areas where you would suggest changes to the narration? |
| Concepts | - Does the video clearly introduce the parenting skills? - Are the skills presented relevant to your family? Why or why not? |
| Goals | - Are the parenting skills consistent with your values like education, family, and morals? |
| Context | - Is the context of forced migration clearly demonstrated in the video through the actors, pictures, scenarios, and narration? |

*Note.* EVM: Ecological Validity Model (Bernal et al., 1995)
